# Letter to Editor: “Lived Experiences of Returning to Participation After Mild Stroke: A Phenomenological Study in Spain”

**DOI:** 10.1111/hex.70959

**Published:** 2026-10-30

**Authors:** Asmi Sharma, Manju Devi

**Affiliations:** ^1^ Department of Physiotherapy Lovely Professional University Phagwara Punjab India

Dear Editor,

We read with great interest the article titled “Lived Experiences of Returning to participation After Mild Stroke: A Phenomenological Study in Spain” by de Diego‐Alonso [1]. We found this study to be a valuable contribution in the field of research as it helps us to percieve the experiences of a population that is mostly overlooked which is stroke survivors with mild sequele. This particular group is often assumed to not undergo any difficulty resuming their daily life along with facing any participation restrictions. The author's phenomenological approach, based on Husserl's framework and Giorgi's method efficiently captures the individual nature of returning to daily life, work and social responsibilities after surviving stroke, even the impressive use of patient's narrative futher strenghthens the qualitative depth of the findings. We highly commend the work done by the respected researchers and merely present our suggestions to further clarify how sample heterogeneity and inclusion criteria may have shaped the reported data collected.

However, with due respect, few aspects of the study require further reflection. Firstly, the sample heterogeneity in time since stroke is questionable. Inclusion criteria of participants consisted of sample span of roughly 15 months to nearly 9 years post stroke. Considering such big gap under one single coherent experience directly influences the reliability of the qualitative data collected. As participation restriction, trajectory of recovery along with other factors differ vastly for someone 1 year post stroke and someone 8 years post stroke [2]. Further inclusion criteria must be more justified as evidently the study concluded that out of 35 participants only six lived alone and 12 required caregiver's assistance. This distorts the baseline and supports the above said claim of comparing unreliable experiences of different groups. Either everyone should have a caregiver or no one in particular. In addition to this caregiver's perspective should also have been included as it would have given more clarity in patient's conditions considering the fact that the study is qualitative.

Secondly, the term “mild” stroke classified for the sample is contradictory. The reason being despite having high Barthel index score of 93/100 and Functional Ambulation Category (FAC) > 3, 12 of the 35 participants required caregiver assistance for daily activities and only six lived alone. This requires scrutiny as it fails to match the functional reality of over one‐third of the sample. Attaining such high scores in functional tests and still being dependent doesn't match the practical outcome of the scores achieved.

Thirdly, the sample distribution in relevance to gender that is 2:1 (23 men, 12 women) is resulting in biasness and should be considered under limitation as sampling imbalance and not be categorised as a finding. The fact the stroke is prevalent in males could be the reasoning for the sample distribution but it should be mentioned in the article itself rather presenting vaguely [3].

Fourthly, the sampling method should me more clarified as initially it is mentioned that purposive sampling method was followed as it's more common in phenomenological studies but further it is said that participants were recruited consecutively. Both of these are different approaches for recruiting sample thereby it needs to be justified as how exactly the sample size was considered for the study.

We appreciate the author's meaningful contribution to grasp the lived experience of mild stroke survivors and look forward to future studies that continue to build on this important line of research.

## Author Contributions

**Asmi Sharma:** conceived the idea and drafted the manuscript. **Manju Devi:** reviewed the literature and critically revised the manuscript. Both authors read and approved the final version.

## Conflicts of Interest

The authors declare no conflicts of interest.

## Data Availability

Data sharing not applicable to this article as no data sets were generated or analysed during the current study.
